# Evaluating new treatments for advanced cancer

**DOI:** 10.1054/bjoc.2001.1887

**Published:** 2001-07

**Authors:** Nicholas Botwood

**Affiliations:** Research Physician Lilly Oncology, ENC – SPC, Sandler, Comelia


doi: 10.1054/ bjoc.2001.1887, available online at http://www.idealibrary.com on IDEAL®

### Evaluating new treatments for advanced cancer

**New treatments for advanced cancer: an approach to prioritization**
**JSJ Ferguson, M Summerhayes, S Masters, S Schey and IE smith**
**Br J Cancer 83: 1268–1273**

**Sir**,
I would like to bring your attention to a number of significant errors in the above article which are extremely misleading, both in terms of the clinical effectiveness and cost of the drugs evaluated. For ease of reference I have listed where mistakes have been made below:

**Table 1** Gemcitabine is also licensed for treatment of advanced bladder cancer (muscle invasive Stage IV tumours with and without metastases) in combination with cisplatinum.

Paclitaxel is also licensed for the treatment of non small cell lung cancer (NSCLC)

Docetaxel is also licensed for the treatment of second line NSCLC.

The table, as it stands, is incomplete and misleading about the licensed indications of these drugs.

**Table 1** The costs have been calculated on a 'per cycle basis'.

The costs of Gemcitabine per cycle is listed as £1030, and the cost of Vinorelbine £175 – suggesting that Gemcitabine is approximately 6 times as expensive as Vinorelbine on a per cycle basis. The actual costs are in fact similar (for a *cycle* or course of treatment) and the table should therefore be corrected, so that *3 infusions* of Gemcitabine are not compared to *one infusion* of Vinorelbine.

**Table 3** The effectiveness of new treatment scale claims that Vinorelbine/Cisplatin has a 3–6 month survival advantage over Cisplatin in first line NSCLC, with strength of evidence alpha +. It would be interesting to see on what data this claim is based. There is no mention of the comparable trial of Gemcitabine/Cisplatin vs Cisplatin – in the first line setting, which should be included here as it showed a significant survival advantage ($P = 0.004$). Added to which 39% one year survival for the Gemcitabine/Cisplatin combination is not surpassed by any similar Vinorelbine/Cisplatin combinations.

It is also worth noting that in the only comparative trial where Gemcitabine/Cisplatin and Vinorelbine/Cisplatin have been compared (although admitedly not compared head to head) the Gemcitabine/Cisplatin arm appeared extremely favourable to the Vinorelbine/Cisplatin arm in terms of survival, where the Vinorelbine/Cisplatin arm was dropped at the interim analysis stage due to inferior efficacy.

Gemcitabine has no licence for the second-line treatment of NSCLC and this statement is therefore incorrect and should be removed, added to which there are no randomized data to support this effectiveness claim in the second line setting.

There are a number of tumour types where new treatments are available that have not been listed in the table such as pancreatic cancer, bladder cancer and glioblastoma multiforme.

These are the major inaccuracies.

It is also worth commenting that whilst survival is often considered the most important endpoint in clinical trials, tumour types which are notoriously chemoresistant such as NSCLC, pancreatic cancer, and renal cancer can be discriminated against by using a generic ranking scale which compares relatively chemosensitive disease such as ovarian cancer, with those where survival benefits may be hard to show, but where improvement in quality of life may be just as important.

**Dr Nicholas Botwood**
**Research Physician Lilly Oncology**
ENC – SPC,
Sandler,
Comelia doi: 10.1054/ bjoc.2001.1890, available online at http://www.idealibrary.com on IDEAL®

### Evaluating new treatments for advanced cancer – reply

**Sir**,
Neither the use of gemcitabine in bladder cancer nor the use of docetaxel in non small cell lung cancer NSCLC were licensed for these indications in the UK at the time of writing or preparing the manuscript. More importantly there had been no demand from clinicians locally to use these products in these indications. This also explains the omission of paclitaxel treatment for NSCLC (licensed Nov 1998). It should be noted that in our next meeting to appraise new anticancer agents we will be considering the use of gemcitabine plus cisplatin in bladder cancer.

### Cost per cycle

Price does not affect a rating a drug receives. We would concede that if used as a price comparison for different treatments Table 1 could be misleading. However it would be extremely unwise to do this for several reasons including the variability in pricing between institutions and over time depending on the level of discount and the variant regimens of the different drugs used.

There is a good reason why the vinorelbine price per week is given. At the time of writing (and currently) the dosage schedules used for this drug are more variable than most and include once weekly continuous treatment – with such a treatment, the appropriate cycle length is very hard to define.

### Relative effectiveness of vinorelbine

There are many trials of vinorelbine in NSCLC that could be considered when appraising its efficacy. Probably the two most relevant are the following. A large trial ($n = 612$) by Chevalier et al

© 2001 Cancer Research Campaign 

